# Unlocking cyanidin-3-glucoside potentials with green technologies: advances in extraction, bioavailability, and stability for therapeutic and non-therapeutic applications

**DOI:** 10.3389/fnut.2026.1830948

**Published:** 2026-07-02

**Authors:** He Zhu, Yang Yu, Otobong Donald Akan, Bo Li, Egong John Egong, Mengliu Zhu, Maria Emmanuel Bassey, Ofonime Emmanuel Udofia, Yanxia Xing, Sheng Liu

**Affiliations:** 1 College of Modern Beef Cattle Industry, Shandong Agriculture and Engineering University, Jinan, China; 2 College of Food Science and Engineering, Central South University of Forestry and Technology, Changsha, Hunan, China; 3 Department of Microbiology, Faculty of Biological Sciences, Akwa Ibom State University, Uyo, Akwa Ibom, Nigeria; 4 Department of Microbiology, Faculty of Biological Sciences, University of Nigeria, Nsukka, Nigeria; 5 Department of Genetics and Biotechnology, Faculty of Biological Sciences, Akwa Ibom State University, Uyo, Akwa Ibom, Nigeria

**Keywords:** cyanidin-3-glucoside, natural pigments, food quality, functional foods, the gut-microbiome-liver-brain-immune system axis

## Abstract

Research exploring and coupling green technologies and the multi-functional cyanidin-3-glucoside (C3G) molecule is increasing due to many reasons. Beyond its role in plant defense, emitting several plants’ hues, and pollination, the unique C3G’s structure supports diverse health benefits (therapeutic) and even photochromic (non-therapeutic) properties. A naturally abundant anthocyanin, carbon-rich C3G molecule is found in pigmented plant parts and is now producible via an engineered *E. coli* strain; however, its numerous applications suffer from its sensitivity to light, oxygen, enzymes, pH, and heat, and the environmental toll of its conventional extraction has limited real-world use. Green techniques are selected due to their low environmental impact, efficiency, and ability to yield by-products that are capable of withstanding harsh environmental conditions. Recent green innovations such as deep-eutectic solvents (DES) are recovering up to 91% of phenolics with 1.5–3 times higher antioxidant activity, while cyclodextrin encapsulation enables the molecule to boost gut microbiome benefits—promoting good bacterial (*Bifidobacterium* spp.) growth and suppressing the growth of harmful bacteria (e.g., *Clostridium histolyticum*) in *in vitro*, animal, and human trial studies. Coupling sustainable green extraction and delivery methods can boost the therapeutic functions of the C3G molecule through the gut-microbiome-liver-brain-immune system axis and enhance its (non-therapeutic) photochromic and additive benefits through improved molecular stabilization.

## 1 Introduction

Green extraction and delivery technologies provide several benefits over conventional methods, including a low environmental impact, improved molecular integrity, enhanced health, and non-therapeutic functions (1, 2). Therefore, the search for and use of processes that improve extraction efficiencies, molecular stability, bioaccessibility, and bioavailability of bioactive molecules are increasing, as they broaden their applications and benefits, with reduced negative environmental impacts (3, 4). Green technologies improve the quality and safety of food-derived products: Research shows that green extraction and stability technologies drastically reduce intestinal absorption and distortion of the cyanidin-3-glucoside (C3G) molecule while improving its molecular stability and solubility (5, 6), making its therapeutic and non-therapeutic applications more effective.

The increasing interest in studies relating to the C3G molecule stems from its relatively cheap and abundant sources and therapeutic and non-therapeutic applications (5, 7–9). C3G, the most abundant anthocyanin in plants, has been widely studied, and research shows its presence and metabolites are among the most consistently observed in human tissues (10, 11). After ingestion, the C3G molecule can remain in the bloodstream for up to 48 h (12, 13). The C3G molecule elicits its multifaceted health functions through the notable gut-microbiome-brain-liver-immune axis (14, 15). However, the complexity of human physiology, genetic variability, and individual gut microflora collectively impact the digestive and catabolic processing of the C3G molecule as well as the rate at which it travels through the gastrointestinal tract and the extent to which it is absorbed or excreted. These factors further complicate its variable bioavailability, metabolic patterns, and heterogeneous uptake (7, 8, 16, 17).

The phenolic hydroxyl group in the C3G molecule is prone to degradation, leading to its low bioavailability and stability, limiting its phytotherapy and pigment-related uses (5, 18, 19). Physical and physiological conditions, including pH, molecular components, enzymes, light, high temperatures, oxygen, and solvents, affect the structural stability and intended functions of the C3G molecule by attacking this hydroxyl group (20–23). Therefore, pertinent reasons for adopting green extraction and delivery technologies for this molecule stem from the increasing demand for plant-based functional foods. This demand will continue as more categories of consumers prefer vegan diets, are allergic to animal-sourced foods, and prioritize health and wellness, and as food companies replace synthetic food additives with safer and nutrient-rich alternatives (7, 8, 16–19, 24, 25). This review compiles literature on plant-based sources, conventional extraction methods, and therapeutic and non-therapeutic functions of the C3G molecule. It sheds light on green methods that enhance the bioavailability and molecular stability of the C3G molecule and, in turn, improve its therapeutic and non-therapeutic functions.

## 2 The C3G molecule: structure, sources, metabolism, and mechanisms

### 2.1 The C3G molecule’s structure and sources

**Figure 1:**
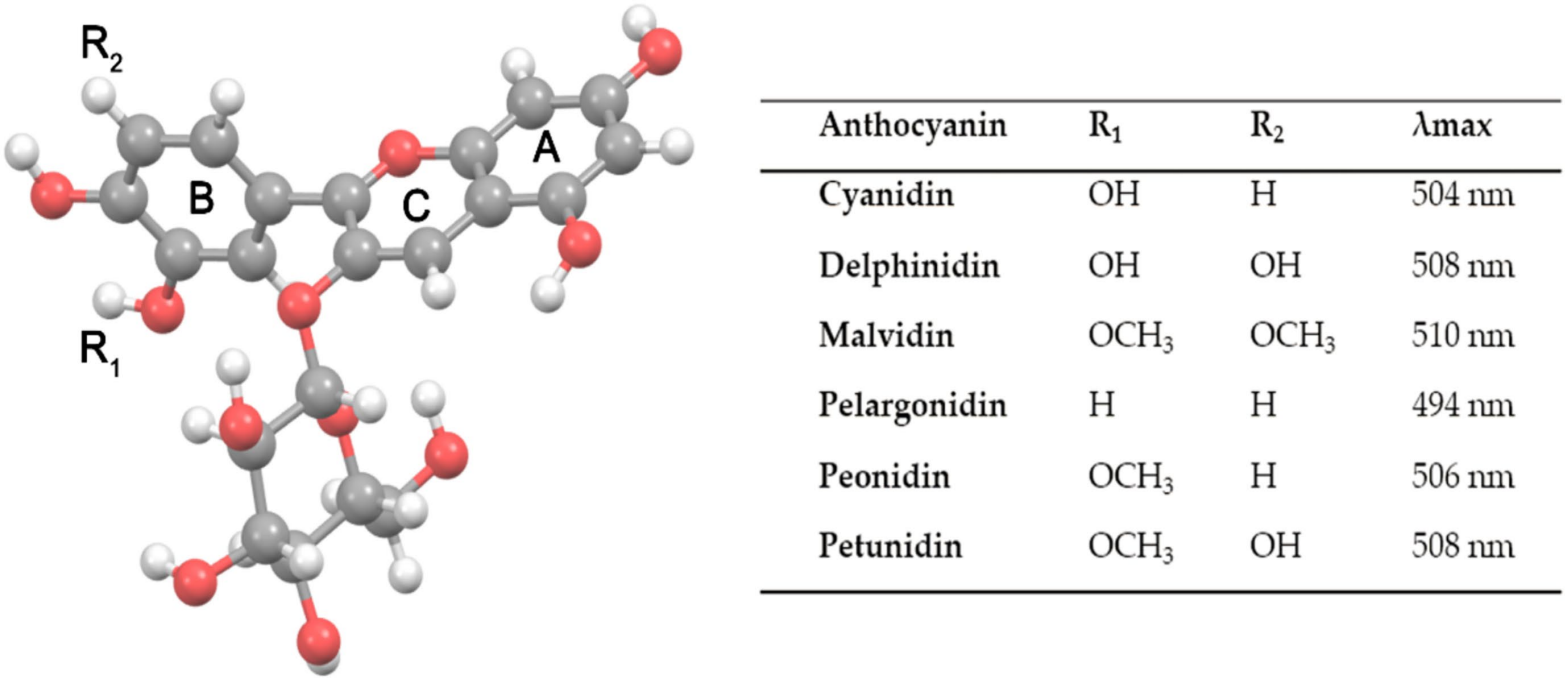
Cyanidin-3-glucoside (C3G) molecular structure. The anthocyanidin (cyanidin or aglycon) consists of an aromatic ring (A) bonded to a heterocyclic ring (C) that contains oxygen bonded by a carbon-to-carbon bond to a third aromatic ring (B).

The primary biological functions of the C3G molecule include expressing a range of cyan color shades or hues, playing a defensive role in native plants (and plant parts), pollination, and structural functions (26, 27), and thereafter, promoting health benefits in humans after digestion (12, 28). This notable water-soluble secondary plant metabolite is known as cyanidin-3-glucoside, cyanidin-3-O-glucoside (C-3-O-G), *asterin*, or *chrysanthemin*. Its unique structure has eight positively charged conjugated double bonds, which are responsible for the pale yellow-to-blue colors of plant parts. The molecule is visibly intense reddish under acidic conditions and bluish under alkaline conditions (29, 30). Its aglycone structure consists of a carbon skeleton containing C-6 (A-ring), C-3 (C-ring), and C-6 (B-ring), as seen in Figure 1. It has a positive charge on the oxygen atom of the C-ring, known as a flavylium ion, and gives the molecule’s ionic character (26, 31). Polyhydroxy and polyethoxy C3G glycosides are derived from flavylium or phenyl benzopyrilium salts (29).

The presence of two hydroxyl groups in the B-ring gives the C3G molecule its high anti-oxidative properties (31), which is associated with numerous positive health-promoting functions. However, as pH increases and hydration proceeds, a colorless carbinol pseudo-base structure and a colorless chalcone are formed (32). The chemical structure of the C3G molecule differs from other common anthocyanins by the composition of its R_1_ (OH) and R_2_ (H) groups and absorbance wavelength (504 nm). The C3G molecule is frequently utilized as a standard for quantifying anthocyanin content extracted from dietary sources and exhibits a reddish to purple coloring at pH 8 (10, 11, 28).

Although detailed information on the C3G content of different foods is scarce, concentrations vary across the outer epidermal peels of some pigmented food items (7, 8, 26, 27, 31). A retrospective study evaluating eight pigmented plants (*Lonicera caerulea* L., *Rubus fruticosus* L., *Ribes nigrum* L., *Morus alba* L., *Zea mays* L. seed, *Z. mays* L. cob, *Brassica oleracea* L., and *Dioscorea alata* L.) found varied (97.11 to 320.27 milligrams) C3G content (33). The study further confirmed a direct correlation between the plant’s pigmentation and anthocyanin content, meaning the darker the plant pigmentation, the higher the anthocyanins’ content and anti-oxidative activity, and possibly higher or better biofunctions. This variable content is affected by genes, light, temperature, and agronomic factors in plants (29). Table 1 presents a compilation of biofunctions of the C3G molecule from plant-based food types, including cereals, grasses, legumes, spices, roots, bulbs, pigmented grains, flowers, tubers, and cole crops (9, 12, 28, 29, 34).

**Table 1:** Some biological functions of food-based cyanidin-3-glucoside (*in vivo* and *in vitro* studies).

Anti-oxidative experiments	C3G function pathway	Reference
C3G from berries in pre-treated HT22 cells.	Scavenged radical activity, inhibited intracellular ROS generation, and suppressed the upregulation of calpain, caspase-12, and CHOP protein levels. Protected cells from glutamate-induced oxidative toxicity, upregulated ERK and Nrf2 expression levels, and drastically activated SODs, CAT, GPx, and phase II enzymes (GSTs) expression levels.	(79)
C3G from *Lonicera caerulea* L. berry in HepG2 cell lines	Attenuated H_2_O_2_-induced cell apoptosis through ROS scavenging.	(74)
C3G from mulberry fruit in MIN6 pancreatic *β*-cells.	Reduced the H_2_O_2_-induced cell death in the MIN6N pancreatic β-cells. Regulated Bcl-2 family, cytochrome C, and caspase-3 and decreased MDA content.	(103)
C3G from blackberry in mice and cultured JB6 cells.	Inhibited UVB- and TPA-induced transactivation of NF-*κ*B and AP-1. Inhibited the expression of COX-2 and TNF-*α*.	(104)
C3G in Korean Rubus fruits in RAW264.7 murine macrophage cells.	Decreased the protein expressions of iNOS and COX-2 in cells treated. Downregulated NF-*κ*B expression and upregulated I-*κ*B expression in LPS-treated macrophages.	(105)
C3G from *Lonicera caerulea* L. berry in C57BL/6 mice.	Alleviated CCl4-induced liver damage through elevating antioxidant enzyme activities and upregulating the Nrf2-antioxidant pathway.	(74)

Anti-inflammatory experiments	C3G function pathway	Reference
C3G from blackberry in mice and cultured JB6 cells.	Inhibited UVB- and TPA-induced transactivation of NF-*κ*B and AP-1. Inhibited the expression of COX-2 and TNF-*α*.	(104)
C3G from black rice in A549-lung cells and differentiated THP-1 macrophages.	Significantly suppressed NLRP3, IL-1β, and IL-18 inflammatory gene expression levels.	(78)
C3G from mulberry fruit in MIN6 pancreatic *β*-cells.	Inhibited JNK, ERK, and p38 phosphorylation.	(103)
C3G from *Lonicera caerulea* L. in mice.	Decreased inflammatory cytokine levels (e.g., IL-1β, TNF-α, C-reactive protein, and IL-6), decreased inflammation-related factors, including COX-2 protein and NF-*κ*B mRNA, and inhibited NF-*κ*B kinase α mRNA expression levels.	(34)
C3G from the black soybean peels in mice.	Inhibited inflammatory cytokines through the blood–testis barrier and inflammatory mediators in the mice serum. Improved DSS-induced impaired fertility	(82)

Anti-obesity experiments	C3G function pathway	Reference
C3G from black beans in differentiated 3 T3-L1 cells.	Increased adiponectin concentration in a differentiated 3 T3-L1 cell in a dose-dependent manner. Significantly decreased TNF-α concentration and ROS production. Promoted adipocyte differentiation and glucose uptake in a dose-dependent manner.	(106)
C3G from purple corn in mice.	Significantly suppressed the high-fat diet-induced increase in weight gain and white and brown adipose tissue weights. Suppressed the mRNA levels of fatty acid and triacylglycerol synthesis enzymes and lowered the sterol regulatory element-binding protein-1 mRNA level in white adipose tissue.	(107)
C3G from dark-red berries in male C57BL/6 J mice.	Attenuated glucose, lipids, insulin resistance, and inflammatory markers. Increased the B/F ratio in male C57BL/6 J mice.	(108)
C3G from various colored edible plant materials *in vivo* models.	Reduces gain in body weight in diet-induced mice by downregulating the mRNA levels of sterol regulatory element-binding protein-1 in white adipose tissue.	(30)

Anti-diabetic experiments	C3G function pathway	Reference
C3G-rich bayberry fruit in β-cells.	Prevents β-cells’ death, improves viability, diminishes reactive oxygen species (ROS) production in the mitochondria, and enhances insulin protein in cells.	(109)
C3G from mulberry fruit in MIN6 pancreatic β-cells.	Prevented diabetes by preventing oxidative stress-induced β-cell apoptosis. Reduced the H_2_O_2_-induced cell death in the MIN6N pancreatic β-cells. Inhibited the phosphorylation of ERK and p38 without inducing the phosphorylation of JNK. Regulated the intrinsic apoptotic pathway-associated proteins (Bcl-2, family, cytochrome c, and caspase-3).	(103)

Cardio-protective experiments	C3G function pathway	Reference
C3G from berries in mice.	Effectively reduced cardiac injury, oxidative stress, and mitochondrial damage following myocardial ischemia/reperfusion. Changed mice’s microbiome and improved the cardio-protection via the transplantation of fecal microbiota of a C3G-enriched diet to mice fed a standard diet. It promotes relaxation of the aorta ring, reduces atherosclerotic lesions, and improves endothelial functions, all of which have cardio-protective benefits.	(80)
C3G from berries, vegetables, parts of plants, and cereals in human intervention studies.	It prevents cardiovascular complications by altering gut microbiota, acts as an antioxidant, and prevents inflammation and other metabolic dysfunctions. These contribute significantly to cardio-protection.	(110)

Anti-cancer experiments	C3G function pathway	Reference
C3G from blackberry in cultured JB6 cells.	Blocked TPA-induced neoplastic transformation in JB6 cells. Inhibited proliferation of A549 cell lines. Decreased ethanol-mediated cell adhesion to the ECM. Inhibited migration and invasion of A549 tumor cells. Inhibited ethanol-stimulated phosphorylation and interactions between ErbB2, cSrc, FAK, and p130Cas proteins. Abolished ethanol-mediated p130Cas/JNK interaction.	(104)
C3G from mulberry fruit in MIN6 pancreatic β-cells.	Inhibited the phosphorylation of ERK and p38 without inducing the phosphorylation of JNK. Reduced the H_2_O_2_-induced cell death in the MIN6N pancreatic β-cells.	(103)
C3G from *Morus alba* L. in MDA-MB-453 human breast cancer cells.	Decreased cell viability, altered apoptotic protein contents, and DNA fragmentation.	(77)
C3G from blackberry in mice.	Decreased non-malignant and malignant TPA-induced skin tumor numbers in mice. Reduced the size of A549 tumor xenograft growth and significantly inhibited metastasis in nude mice.	(104)
C3G from *Lonicera caerulea* L. in mice.	Increased tumor apoptosis and downregulated metastasis-related factors, such as transforming growth factor-β, CD44, epidermal growth factor receptor, and vascular endothelial growth factor. Altered the expression of the tumor microenvironment-related factors Ki67, CD45, PDL1, and CD73. Regulated the intrinsic apoptotic pathway associated with the Bcl-2 family, cytochrome c, and caspase-3 proteins.	(34)
C3G from *Morus alba* L. in a xenograft animal model.	Significantly reduced tumor growth in xenograft nude mice. Induced apoptosis via the Bcl-2 and Bax pathway.	(77)

Neuroprotective experiments	C3G function pathway	Reference
C3G from black currant and berries in microglia (BV-2) and cardiomyocyte (HL-1) cell lines.	Reduced oxidative stress in BV-2 and HL-1 cells.	(81)
C3G in berry fruits in HT22 hippocampal neuronal cells.	Inhibited glutamate-induced oxidative and ER stress signal and activation of ERK/Nrf2 antioxidant mechanism pathways.	(79)
C3G from the mulberry fruit (*Morus alba* L.) in rat primary cortical neurons.	Prevented membrane damage and preserved mitochondrial function of the primary cortical neurons exposed to OGD in a dose-dependent manner. Preserved mitochondrial membrane potential (MMP) in primary cortical neurons exposed to OGD. Enhanced osteoblast proliferation rate and osteoblast mineralization points.	(111)
C3G from tart cherries in pMCAO C57BL/6 J mice.	Significantly improved neurological functions and lowered brain superoxide levels in protected mice. Attenuated infarction volume and blocked the release of AIF from mitochondria under oxidative stress.	(112)
C3G in female heterozygous AD mice, B6C3-Tg and non-transgenic mice (C57BL/6Jms).	Decreased the levels of amyloid-beta (Aβ) peptides and reduced the protein expression of amyloid precursor protein (APP), presenilin-1, and β-secretase in the brain regions. Improved autophagy in the brains of mice by upregulating the protein expression of autophagy-related markers, such as LC3B-II, LAMP-1, TFEB, and PPAR-α. Regulated the PI3K/Akt/GSK3β signaling pathway, which could inhibit the phosphorylation of tau and thus improve synaptic function and plasticity.	(113)

Anti-aging experiment	C3G function pathway	Reference
C3G from fruit and vegetable sources in *Caenorhabditis elegans.*	Extended the lifespan of *C. elegans* by 14% and enhanced the pharyngeal pumping by 6.3% without stress. Improved the pharyngeal pumping rate of the nematode by 6% under UVA stress. Prolonged the lifespan of *C. elegans* by 62% and augmented the pharyngeal pumping by 6.2%.	(114)

Visual improvement experiments	C3G function pathway	Reference
C3G from black rice in human retinal endothelial cells (HREC).	Efficiently prevented retinal endothelium barrier dysfunction due to high glucose.	(115)
C3G from dried and juiced bilberries in C57Bl/6 mice.	Blocked renal apoptosis and ERS protein expression in renal ischemia/reperfusion (I/R) injury.	(116)
C3G from blueberries and strawberries in adult women.	Long-term intake slowed or delayed cognitive decline by up to 2.5 years.	(117)

Reproductive experiments	C3G function pathway	Reference
C3G from the black soybean peels in mice.	Improved colitis impaired fertility by reversing DSS-induced colitis, low sperm count, low testis weight, high inflammation levels, and abnormal thickness of the seminiferous epithelium. Stabilized body weight, longer colon length, less crypt damage, and focal inflammation infiltration.	(82)
C3G from black rice in mice.	Prevented liver fibrosis progression in mice induced by carbon tetrachloride.	(118)
C3G from the black soybean peels in mice.	Improved DSS-induced impaired fertility	(82)

Prebiotic/anti-microbial experiments	C3G function pathway	Reference
C3G from hawthorn fruit in microbial cultures.	Inhibited the growth of *Escherichia coli, Pseudomonas aeruginosa, Salmonella abony,* and *Candida albicans.*	(90)
C3G from black rice in A549-lung cells and differentiated THP-1 macrophages.	Significantly inhibited spike glycoprotein S1 subunit of SARS-CoV-2 (SP)-induced inflammatory responses for both gene and protein levels.	(78)
	Attenuated SP-induced inflammation via counteraction with NF-*κ*B activation and downregulation of the inflammasome-dependent inflammatory pathway proteins (NLRP3, ASC, and Caspase-1).	
C3G from Peruvian purple corn.	Promoted the growth of beneficial probiotic lactic acid bacteria such as *Lactobacillus helveticus* and *Bacillus longum.*	(119)
C3G from *Vaccinium meridionale* Swartz berries.	Inhibited the growth of *Staphylococcus aureus* (more) and *Escherichia coli* (less).	(91)
C3G from dark-red berries in male C57BL/6 J mice.	Partially neutralized HFHS diet-induced alterations of gut bacteria. Increased the B/F ratio in male C57BL/6 J mice.	(108)

Non-therapeutic functions	C3G function	Reference
Food ingredients		
C3G from *Arbutus unedo* fruits in wafers.	Sustained natural coloration and extended shelf life of wafers after elongated storage.	(87)
C3G from barberry (*Berberis vulgaris*).	Increased the sensory attributes, color, and shelf life of jelly powder during storage.	(86)
C3G in rice-based bakery products.	C3G-enriched black rice (25%) was used to fortify red or white pericarp rice (75%), yielding a better nutritional quality for cooking and baking, evaluated through mineral profiling, UFLC, and LC–MS.	(120)
Cosmetics		
C3G gel was used on 4-week-old female Kunming (KM) mice.	Reduced chronic photodamage caused by UVB by inhibiting oxidative stress and inflammation. Effectively ameliorated the UVB-induced epidermal barrier dysfunction, including increased skin hydration and decreased transepidermal water loss. Inhibited UVB-induced epidermal hyperplasia, the destruction of collagen fibers, ROS levels, and the expression of COX-2 and IL-6.	(121)
Green power generation		
Dye-sensitized solar cells using C3G extracted from crushed leaf stocks of *Manihot esculenta Crantz* and *Eugenia Jambolana* (Indian blackberry, locally called Naval).	Increased electron density in the conduction band of TiO_2_ (pH 2 to 10). Effectively attached to the surface of the TiO_2_ film and exhibited a short-circuit photocurrent of 1.49 mA and a power conversion efficiency of 0.5%.	(95, 96)
C3G extract from Gayo Arabica coffee husk	Through a computational model, the highest occupied molecular orbital (HOMO) and the lowest unoccupied molecular orbital (LUMO)—the energy gap for C3G and C3R molecules is 3.37919316 eV and 0.28792381 eV, respectively.	(97)

### 2.2 The C3G molecule’s metabolism and mechanisms

**Figure 2:**
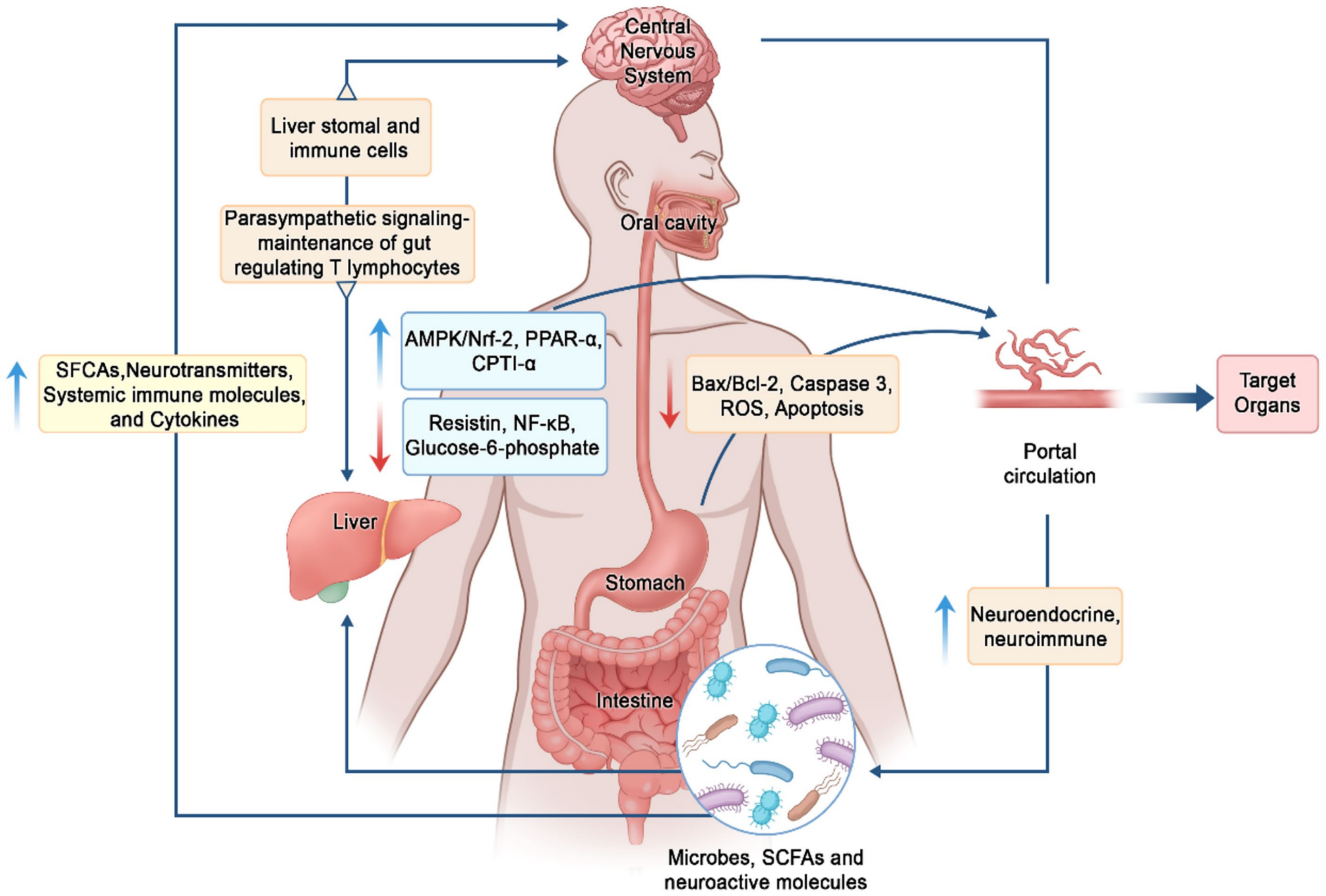
Gut-microbiome-liver-brain-immune system axis metabolism mechanism of the cyanidin-3-glucose (C3G) molecule. A summary of some avenues that overlap interactions and actions of C3G and its metabolites via organs involved with its metabolism, absorption, and transport. The C3G molecule metabolism moves from the mouth, stomach, small intestine, large intestine, systemic circulation, to the target organs. The gut microbiome plays a vital role in metabolizing a large proportion of C3G into functional metabolites.

The combined action of the C3G molecule and its metabolites is thought to be responsible for the health-promoting functions they elicit (7–9). However, the stability of the C3G molecule’s structure and its fate during ingestion significantly impacts its bioavailability and activity in target tissues (7–9, 16). The metabolism of the C3G molecule starts in the mouth (see Figure 2). During chewing, the C3G molecule is decomposed by enzymolysis (enzymes in saliva)—its aglycones become more lipophilic and are easily absorbed (35). In the oral cavity, *β*-D-glucosidase and β-D-galactosidase in saliva help break down the C3G molecule to protocatechuic acid (PCA) and phloroglucinaldehyde (PGA). Interestingly, the microorganisms in the oral cavity and the gut are partly similar (35). The C3G molecule gets to the gastric epithelial cells through active diffusion and the aid of transporters (36).

In the stomach, a significant proportion of the C3G molecule is absorbed unhydrolyzed (by gastric epithelial cells by active diffusion). It is speculated that the C3G molecule is very stable in the stomach at low pH (1.3 ± 0.2) (35). However, UDP-glucuronosyltransferase in the gastric wall metabolizes some flavonoids into glucuronidated and sulfated metabolites. The C3G molecule is instantly submerged in the alkaline environment of the small intestine, where it breaks down and becomes hemiketal and chalcone. A chamber experiment showed that the C3G molecule is primarily absorbed in the jejunum (accounting for 55%), only approximately 10% in the duodenum, and almost none in the ileum and colon (35). In addition, the absorption rate of the methylated C3G molecule in the gut and liver is drastically reduced (37). The absorption of the C3G molecule is impacted by a pH of between 7.5 and 8.0, the pancreatic juice, and brush border enzyme action. The sodium-dependent glucose transporter (SGLT1), glucose transporters (GLUTs 1–3), bilitranslocase transporter (BTL), and mono-carboxylated transporter 1 (MCT1) are essential in promoting the entrance of the C3G molecule into the enterocytes (due to the B-ring and glucose components) (35, 36, 38).

The C3G molecule is hydrolyzed in the small intestine to form aglycones (27). The large intestine is the next port of call. It contains a highly diverse microbial population (10^12^ microorganisms per gram of gut contents). The large intestine is crucial to the metabolism of C3G, since 90–99% of ingested C3G that is not absorbed in the small intestine eventually reaches the colon, where the gut microbiome is responsible for enzymatically converting the molecule through deconjugation and dehydroxylation (38). Processes modulated by gut microbes and their enzymes cleave the heterocyclic flavylium ring (C-ring) of the C3G molecule to produce phenolic compounds delivered to the circulatory system (12, 27, 35). In mice studies, the C3G molecule upregulated the floral abundance of *Bacteroidetes, Muribaculaceae, Romboutsia*, and many SCFA-producing bacteria species such as *Ruminiclostridium*_9. On the other hand, it reduced the floral abundance of intestinal *Firmicutes* and *Lachnospiraceae* in mice (31, 39). The bidirectional interaction between C3G and gut microbiota in the colon supports immune modulation—C3G promotes beneficial microbial growth, while microbial metabolites of the C3G molecule enhance gut barrier integrity and immune regulation (35).

Some essential reactions of the C3G molecule occur in the liver cells. Methylated C3G molecule reversibly produces 3-methyl-cyanidin-3-glucoside and 4-methyl-cyanidin-3-glucoside in liver cells (35). Glycosylation and deglycosylation also occur, reversibly converting cyanidin to cyanidin-3-glucoside and *vis-à-vis*. Conversely, the methylated C3G molecule can be spontaneously degraded to generate vanillic acid, protocatechuic acid, and phloroglucinol aldehyde (which could further be metabolized) (35). C3G aglycones are subjected to phase I (demethylation and hydrolysis) and phase II (glucuronidation, sulfation, and methylation) metabolic transformations by lactase phlorizin hydrolase, UGTs, cytochrome P450 1A (CYP1A), sulfotransferases (SULTs), and other likely enzymes (40). Researchers have confirmed that the metabolized C3G molecule can be detected in cardiac and skeletal muscles, liver, eyes, gut, kidneys, and brain, in proportion to the C3G molecule’s concentration in the plasma (35, 38).

## 3 Conventional and green technology extraction methods

### 3.1 Conventional extraction methods and limitations

Extraction procedures are fashioned to reduce diffusion rate, solubility, and equilibrium of the desorbed compounds in the sample matrix, and extraction mode (static or dynamic) conditions influence the amount of extractable polyphenols from a compound (41). Available evidence moderately suggests that the extraction method may significantly influence absorption, stability, and overall health benefits of the C3G molecule. A study comparing extraction methods for blackberry cultivars’ anthocyanin (puree-derived and powder-derived) content observed similar anthocyanin levels; however, the puree-derived anthocyanin extracts demonstrated greater phenolic content, increased total antioxidant capacity, and significantly enhanced cytotoxicity across three cancer cell lines (HT-29, MCF-7, and HL-60) (42).

Aside from its effect on yields, it is well known that conventional extraction methods—such as conventional solvent extraction (CSE) techniques like Soxhlet extraction, percolation, maceration, agitation, and leaching—are also constrained by excessive time, energy, high temperatures, and the use of polluting solvents (41). Conventional extraction methods also require multiple steps, are associated with high costs, contribute to lower yields, and lead to inefficient isolation of the pure C3G molecule. The influence on diminishing bioavailability is shown in a study involving adults who consumed 50 mg of polyphenols in various forms (pure chemical, plant extract, or whole food/beverage), found plasma polyphenol concentrations of approximately 0–4 μmol/L. At the same time, the study showed that approximately half (43%) was excreted, showing low bioavailability (8, 17). This study infers the need for sustainable green delivery technologies as the C3G molecule, like most other plant-based bioactive compounds, encounters biological barriers in target organs/tissues and environmental factors that make it highly degradable, limiting its effectiveness (5, 18).

The molecule’s instability is further exemplified in a study where authors recovered a stable isotopically labeled (^13^C_5_) anthocyanin tracer in the urine and breath of participants and estimated the bioavailability of C3G in humans to be approximately 12% (12). This estimate indicates that a relatively small proportion of the ingested C3G molecule is absorbed into the body. This could be due to its decreasing stability at neutral and alkaline pH conditions, according to a study (43). Another research result shows that low concentrations of parent anthocyanins result in diminished effects *in vivo* than *in vitro* observations (7). Hence, the need for improved extraction and delivery methods if the C3G molecule’s functions are to be intensified.

### 3.2 Green technologies adopted for extraction

Comparatively, green extraction technologies are more sustainable, efficient, and environmentally responsible and improve the stability and bioavailability of the extracted C3G molecule (41, 44, 45). The effectiveness of the procedures and the selection of environmentally friendly solvents are two notable benefits. For example, water’s dipole moment and high hydrogen bonding capacity elude water–water and water-to-biomolecule interactions (46), making it suitable for the polar C3G molecule. Additionally, certain green methods use non-food plant residues during extraction to ensure repeatable results. These sustainable advantages increase the attractiveness of technologies that have an overall positive impact on climate change. Since they are eco-friendly and safe and utilize non-toxic solvents and procedures, the resulting extractant mixtures can be used directly as food, cosmetics, and pharmaceutical additives (41, 45, 47) (see Table 2).

**Table 2:** Green-assisted extraction techniques.

Extraction technology	Procedure	Cost and technology scalability	Remarks	Reference
Deep-eutectic solvent (DES) extraction or natural deep-eutectic solvent (NADES)	Utilizes low-cost and biodegradable liquids made by mixing natural hydrogen-bond donors (HBDs) and acceptors (HBAs) at low temperatures, lowering their melting point (−69 to −149 °C) so they remain liquid at room temperature. They are used to extract bioactives from pretreated plant material, followed by filtration or centrifugation for separation.	Low-cost and easy to prepare, but recovery rates and precise formulations may raise operational costs for sensitive or specialized uses. This method is highly scalable using cheap, common components such as choline chloride and lactic acid but needs optimization—different formulations vary in efficiency based on the target compound.	The filtrate contains bioactive compounds, which can be used directly in food since GRAS-listed DES may boost nutritional value. DES extracted 91% of phenolics and showed 1.5–3 times higher antioxidant activity than conventional methods in millet.	(44, 45, 122)
Enzyme-assisted extraction (EAE)	Utilizes hydrolytic enzymes (such as cellulases, pectinases, and hemicellulases) that break down plant cell wall polysaccharides, disrupting the matrix to release trapped metabolites.	Despite high costs, enzyme specificity cuts waste and aids processing—recycling or immobilized systems can lower expenses, especially industrially. Easy to scale due to low energy needs and broad substrate use, but enzyme specificity limits reaction control at larger scales.	Enzyme-assisted extraction improves metabolite yield, as seen in finger millet with a 28% rise in polyphenols and 1.3-fold lower tannins. Effectiveness depends on enzyme type, dose, and incubation time.	(44, 46)
High voltage electrical discharge (HVED)	Utilizes cavitation to break down plant material and enhance the release, but excessive energy may generate radicals that degrade sensitive metabolites.	High upfront costs for specialized electrical systems, but efficient, high-yield processing can lower per-batch expenses. Scaling is challenging due to high electrical demands and customized reactor needs.	HVED extraction can boost phenolic yield—up to 35% more from grape stems—but energy levels must be carefully optimized as lab-scale settings may not translate to industrial applications.	(46, 123)
Homogenizer-assisted extraction (HAE)	Utilizes high-speed mechanical cutting to disrupt plant cell walls, enhancing the release of intracellular metabolites.	Cuts costs by reducing solvents and steps but needs high-pressure equipment and energy. Scalable with water-based systems at high temp/pressure, and simple reactants make large-scale use feasible.	High shear extraction replaces heat, reduces solvent use, and speeds up processing, offering scalable efficiency. With glycerol enhancing polarity, HVED extracted 539.4–1598.1 mg C3G/kg dm of anthocyanins from grape pomace.	(46, 124)
Microwave-assisted extraction (MAE)	Applied electric and magnetic fields provide oscillating forces that increase extraction efficiency by causing internal friction and localized heating in samples and solutions.	Low operating costs due to less solvent and energy, but high upfront infrastructure investment. Highly scalable due to energy efficiency, quick extraction, and potential for continuous-flow setups.	Microwave-assisted extraction reduces environmental impact, works well with moist plant material, and doubled 3-deoxyanthocyanidin yield (3.1 mg/g) from sorghum hull vs. conventional methods—although heating uniformity requires careful control.	(46, 49, 125)
Ohmic heating-assisted extraction (OHAE)	Pulsed electric fields heat samples and make plant cell membranes more permeable by turning internal water, salts, and acids into conductive pathways, enhancing metabolite release.	High upfront cost, but low operating expenses, efficient heating, and minimal energy loss make it appealing. Highly scalable and easily adjustable with little design change.	This rapid, energy-efficient method ensures uniform extraction with minimal solvent use, preserving heat-sensitive compounds. It yielded 3.28 mg/g DW gallic acid from grapes—higher than the conventional 2.84 mg/g.	(46, 126, 127)
Pressurized hot water extraction (PHWE)	Pressurized hot water extraction uses temperature-controlled water to desorb compounds from plant matrices via diffusion and thermodynamic partitioning, enabling efficient, solvent-free elution.	High initial equipment cost but cuts solvent use and waste treatment, boosting appeal for green industries. Highly scalable due to advances in pressure-resistant equipment.	Water is a safe, eco-friendly solvent, but additives can alter superheated water’s properties. Phenolic solubility in water increases exponentially with temperature.	(41, 128)
Pressurized liquid extraction (PLE) or pressurized solvent extraction (PSE)	Elevated temperature and pressure enhance solubility and mass transfer, enabling efficient extraction with solvents such as methanol or water—commonly known as ASE or PFE.	Scalable via modular continuous-flow systems, although high pressure and heat may affect sensitive compounds. Fairly scalable with modular, continuous-flow design, but high pressure and temperature can challenge sensitive compounds.	Pressurized liquid extraction (PLE) cuts solvent use by 90% and time by two-thirds, while boosting anthocyanins 4 times and phenolics 3 times versus conventional methods—despite lower recovery of heat-sensitive compounds.	(41, 44, 46, 51)
Pulsed electric field extraction (PEFE)	Low-energy pulsed electric fields temporarily destabilize cell membranes via polarization, enhancing permeability and metabolite release.	Low operating energy costs, but high upfront investment due to advanced electronics for pulsed fields. Highly scalable due to fast processing, low energy use, and continuous high-volume capacity.	Strong electric fields can irreversibly disrupt cells, enabling high C3G yields (92.59 mg/g) and antioxidant activity (50%) from rice, with minimal heat damage. While promising, lab-scale PEFE may not directly translate to industrial use.	(44, 46, 122)
Supercritical fluid extraction (SFE)	Supercritical fluids (e.g., CO₂ and water) act like liquids as solvents but with gas-like diffusion, enhancing solute mass transfer during extraction.	High upfront cost for specialized equipment limits use but low operating costs—due to cheap, recyclable CO₂—make it viable where quality justifies expense. Easily fits continuous, batch, or multistage processes for industrial use but needs precise pressure/temperature control, complex systems, and strict safety measures.	SFE reduces solvent use and, with co-solvents, improves extraction—yielding 2.5 times more sterols from foxtail millet than conventional methods, with enhanced oil stability due to high tocopherols and low peroxide value, although extraction may take over an hour for complex matrices.	(41, 44, 51, 129)
Ultrasound-assisted extraction (UAE)	Ultrasound cavitation disrupts plant matrices through shockwaves and microjets, enhancing solubilization by breaking cells, reducing particle size, and improving mass transfer.	Cost-effective for small scale with less solvent, time, and energy—but larger industrial use can raise costs due to higher electricity needs and probe adaptation challenges. Easily scaled from lab to mid-size production, but uneven ultrasonic wave spread limits large-scale use, especially with non-uniform materials.	UAE is a fast, simple, non-destructive method where OH- radicals from cavitation break down extracts. It produced 28.7% more phenol from red sorghum than traditional solvent extraction.	(41, 44, 51, 130)

Bio-derived solvents from renewable plant and aquatic biomasses, including algae and compatible industrial wastes, possess non-toxic and low-viscosity characteristics that make them good extractants for certain bioactive compounds (46). Bio-ethanol is generally regarded as safe (GRAS) because of its high purity and biodegradability; however, due to flammability, it is advised to always mix with water (best at 70:30) (46). In addition, a high percentage of ethanol reduces the solubility and may denature C3G molecules (46). Glycerol is a widespread, non-toxic, non-flammable, non-volatile (under normal atmospheric pressures), and biodegradable alcohol compound produced by various means, including microbial fermentation (46). Studies show that using glycerol (from 50% and above) as an alternative solvent yields efficient extraction of C3G due to the lowering of the dipole moment of the extraction mixture (46). Deep-eutectic solvents (DES) are a new class of natural, highly selective, safe, and biodegradable ionic liquid analog compounds obtained by complexing a quaternary ammonium salt with a metal salt or hydrogen bond donor. Used in DES extraction methods, these customized solvents are useful in effectively recovering both polar and non-polar natural compounds (45, 46). However, there are reports of complex compound toxicity and cytotoxicity compared to individual components (46). Therefore, there is a need for caution when compounding the complex.

A novel biotechnology-based study successfully utilized a synthetic biology technique in *E. coli* BL21 (DE3) to sustainably produce the C3G molecule. The researchers utilized tailored promoters to redirect the metabolic pathway of *E. coli* to generate UDP-d-glucose, a key precursor for C3G synthesis. This synthetic vector system will offer an efficient, eco-friendly alternative for large-scale C3G production in the near future (48). The engineered *E. coli* system effectively generated C3G without requiring exogenous UDP-d-glucose, making it a viable process for the synthesis of other high-value chemicals. To ensure sustainable and cost-effective solutions, adoptable green extraction methods must be evaluated not just for performance but also for production costs, scalability, and environmental impact (2).

To scale substance extraction to profitable commercial quantities, important aspects of green extraction technologies must be considered. Considerations and method adoptions would depend on material characteristics, cost-effectiveness/capital investment, energy consumption/requirement (kWh/kg extract), water usage (L/kg), carbon emission (kg CO₂-eq/kg), and operational efficiency (49). For example, considering the amount of solvent, extraction time, and cost of equipment, the microwave-assisted extraction (MAE) methods require a low-to-moderate amount of solvent, a short extraction time, and less waste generation, although needing specialized equipment (46, 50). Increasing MAE power increases extraction potential as microwave power enhances solvent penetration into the plant matrix. The pressurized liquid extraction (PLE) method uses certain temperatures and pressures to extract natural compounds and requires a moderate amount of solvent and a short-to-moderate extraction time, although needing costly sets of equipment (46, 50). The supercritical fluid extraction (SFE) method uses (reusable) CO_2_, a moderate extraction time, and costly sets of equipment (50). The ultrasonic-assisted extraction (UAE) method is among the best technologies from an environmental point of view (46). This method requires a low amount of solvent and a short extraction period but is limited to small applications (50). With the UAE, extraction time is inversely proportional to ultrasonic power.

Although initial costs of some of these methods might seem substantial, operating at commercial or large-scale can offset expenses through increased efficiency (51, 52). Faster processing and higher yield recovery also contribute to long-term cost savings, making the high cost of equipment worthwhile for high-volume C3G production. Some of these have been outlined in Table 2.

### 3.3 Green technologies adopted to improve bioavailability and stability

Characteristics, such as its electrophilic, nucleophilic, and electron-donating qualities, as well as the creation of new pigments, make the C3G molecule inherently reactive (19, 53). This results in an unstable molecule: a challenge or limitation that hinders its health-promoting benefits and its use as a colorant, as off-colors and odors resulting from oxidation reactions are a serious quality concern (5). Enhancing the stability of the C3G molecule has been a significant concern in many studies (18, 32, 54), with remarkable improvements (37). Green extraction and delivery technologies can increase the amount of the C3G molecule released from the plant matrix during digestion (bioaccessibility) and the fraction that is finally absorbed into the system to elicit its multifarious physiological bioactivities (5, 18, 55). Green stabilization and delivery technologies bypass destabilizing factors such as pH, temperature, light, degradative interaction with other dietary compounds, and the action of active gut microbiota (5, 18, 27, 56–58). For example, co-pigmentation and encapsulation are two promising techniques proposed to overcome the limitations of C3G’s bioavailability and stability. The co-pigmentation process enhances the color intensity of the C3G molecule and involves vertically stacking the C3G molecule and other pigments (called co-pigments). This stacking is accompanied by a bathochromic shift (change in wavelength of light absorption) and a hyperchromic effect (enhancement of color intensity) (32, 59, 60). The co-pigmented C3G molecule does not form a colorless hemiketal structure, meaning the blocking of hydration of chromophores stabilizes the molecule.

Co-pigmentation can be achieved through intra-molecular (acylating two or more acyl-containing anthocyanins), intermolecular (conjugating with other flavonoids and related chemicals), or self-association, depending on the nature of the co-pigment(s) used (29, 59, 61). Nano-encapsulation of the C3G molecule improves its molecular stability, bioavailability, and controlled release properties (62, 63). Encapsulation systems involve entrapping the C3G molecule within a protective matrix, such as liposomes, emulsions, or biopolymers. Various encapsulation techniques, including physical, physicochemical, and chemical techniques, have overcome stability and bioavailability limitations of numerous compounds (5, 35). Encapsulation systems shield the C3G molecule from the harsh digestive environment and increase its bioactivities (64). Nano-encapsulated C3G molecules improved cellular uptake and antioxidant activity, thereby expanding their potential applications in the food, pharmaceutical, and cosmetic industries (5, 58). An example is the sodium alginate-C3G-nanoliposome (SA-C3G-NL), which was noted to protect the C3G molecule from the gut environmental factors that limit its bioavailability. Table 3 shows other recent innovative technologies used to improve/increase the bioavailability and stability of the C3G molecule. These emerging approaches control the kinetics and pH parameters of the C3G molecule (37, 65), thus mitigating the degradation of the C3G molecule’s structure and color. Additionally, compound complexation, enzymatic acylation, and fermentation have been used to increase the physiological bioactivities of the C3G molecule (64, 66, 67). Interestingly, a much stabilized C3G molecule will elicit quality and safer non-therapeutic photochromic and additive benefits (57).

**Table 3:** Emerging technologies for improving bioavailability, stability, and functions of cyanidin-3-glucoside.

S/N	C3G source	Technology	Outcome	Reference
1	Barberry (*Berberis vulgaris*) extract	Spray-dried formulations using maltodextrin alone or combined with gum Arabic or gelatin were optimized for performance—each ingredient enhancing key properties like stability, solubility, and encapsulation efficiency.	Encapsulated C3G (7%) as a natural colorant in jelly powder boosts color stability and shelf life—outperforming synthetic dyes in hue, moisture control, and texture, while improving acidity, ash content, and overall quality.	(86)
2	*Solanum melongena* L. bark	Gum Arabic was effectively used as a carrier material in spray drying, thereby encapsulating and preserving the desired compounds.	Yoghurt with encapsulated C3G maintained stable color and antioxidant activity during storage, while non-encapsulated samples showed declining radical scavenging ability. Surprisingly, phenolic content increased—from 225.17 to 291.98 mg GAE/100 g.	(131)
3	Crabapple (*Malus prunifolia Willd. Borkh*)	A C3G-encapsulated nanoparticle formulation was developed by combining carboxymethyl chitosan and calcium chloride (CMC-CaCl2) to form a stable nanostructure.	Encapsulated C3G (10–40 μmol/L) protected ovarian cells over 24 h by reducing oxidative stress, preventing cytotoxicity and cell damage, boosting progesterone production, and enhancing key steroidogenic markers such as cAMP, 3ß-HSD, and StAR protein.	(132)
4	Mulberry polyphenolic extract (MPE)	Incorporating MPE into a κ-carrageenan-based film resulted in an enhanced biopolymer material.	Adding mulberry polyphenol extract (MPE) to κ-carrageenan film creates a smart, pH-sensitive packaging that shifts color—from red to gray—as pH rises. With 2–4% MPE, the film gains strength, heat resistance, and antioxidant power and successfully detects milk spoilage by color change, making it ideal for intelligent food packaging.	(133)
5	*Jaboticaba* peel extract (JPE)	C3G was effectively integrated into a carrageenan biopolymer matrix enriched with *Jaboticaba* peel extract (JPE).	C3G-enriched carrageenan films (50–100% w/w) show enhanced antioxidant activity, block *E. coli* growth, and shift color with pH—going from purple to brown—while improving opacity. These features make them promising as natural, smart packaging that monitors food freshness and safety.	(89)
6	Casein/C3G (Cs-C3G) nanoparticles	C57BL/6 mice on a high-fat diet were supplemented with Cs-C3G to investigate its potential metabolic benefits.	Cs-C3G (0.01 mL/g BW/day) ameliorated HFD-caused fat accumulation and liver oxidative stress, restored eubiosis, and inhibited the growth of some opportunistic pathogenic bacteria.	(134)
7	C3G liposome nanoparticles	C3G was encapsulated in liposomal nanoparticles and tested on Caco-2 cells, a model for intestinal epithelial cells.	C3G and C3G liposomes (0.20–0.25 mg/mL) reduced mitochondrial activity and viability in Caco-2 cells, with liposomes causing structural changes in mitochondria, lipid droplets, and physalides. They significantly suppressed tumor cell proliferation, highlighting their potential as a targeted therapy in cancer treatment.	(135)
8	C3G nanoparticles developed by ionic gelation.	A C3G-chitosan and sodium tripolyphosphate (TPP) nanoparticle formulation was evaluated in a mouse model of UVB-induced skin photodamage.	Nano-C3G (288 nm, 30 mV, ~45% entrapment) shows rapid, pH-dependent release and strong skin protection against UVB damage. It reduces oxidative stress, suppresses key apoptosis markers (p53, Bax, caspases), and improves skin hydration, structure, and cell survival in UV-exposed tissue—making it a promising candidate for dermal photoprotection.	(64)
9	C3G liposomes via the ethanol injection method	C3G encapsulated in liposomes was investigated for its protective effects against oxidative stress in human gastric epithelial GES-1 cells.	C3G liposomes (75% encapsulation) boosted antioxidant capacity and reduced MDA, effectively protecting human GES-1 cells from H₂O₂-induced gastric damage. This highlights their potential for improving stomach-targeted therapies and enhancing mucosal protection.	(136)
10	Cyclodextrin-encapsulated cyanidin-3-glucoside, delphinidin-3-glucoside and malvidin-3-glucoside	The stability and release of encapsulated anthocyanins were monitored in a fecal slurry environment over 24 h.	*In vitro*, animal, and human trial studies showed that traces of C3G significantly proliferated *Bifidobacterium* spp. and inhibited *Clostridium histolyticum*. Shows direct dietary C3G intake and potential gut health benefits.	(137)
11	Sodium alginate-modified C3G nanoliposomes (SA-C3G-NL) in 3D Caco-2 spheroid model	Combining nano-encapsulation and natural polymer to boost the uptake and delivery of the C3G molecule.	SA-C3G nanoliposomes enhance intestinal barrier function by upregulating tight junction proteins (ZO-1, occludin, and claudin-1) and nutrient transporters (SGLT1 and GLUT2) while reducing efflux via BCRP—improving absorption and offering new insights into targeted delivery using 3D gut models.	(58)
12	Red raspberry with five phenolic acids as co-pigments	A blend of phenolic acids was evaluated for its overall synergistic oxidative capacity and health benefits.	Co-pigmentation of C3G and Cy-3-soph with ferulic acid (molar ratio of 1:100 at pH 4) enhanced the color intensity of C3G and enhanced the bathochromic shift and hyperchromic effect at *k*max.	(60)
13	Black rice fiber	C3G and rice dietary fiber underwent co-fermentation to explore their combined effects on gut microbiota and metabolic health.	Combined C3G and insoluble dietary fiber (IDF) fermentation bio-transformed C3G into phenolic compounds with stronger antioxidant activities, increased total SCFA production, modulated the microbiota structure, and bloomed Bacteroidota and Prevotellaceae-related genera.	(138)
		High-pressure technology was applied to co-ferment C3G with RG-I pectin, a soluble dietary fiber.	Twenty-four (24) h HPP-treatment of C3G-RG-I pectin significantly decreased pH from 7.03 to 5.59, promoted short-chain fatty acids production (acetic acid and butyric acid), promoted a richer gut flora composition, promoting the growth of potentially beneficial genera (i.e., Faecalibacterium, Parabacteroides, and Bifidobacterium).	

C3G’s color stability directly impacts its performance as a versatile photochromic pigment in many sectors, including pharmaceutical products, food coloring, cosmetics, solar cells, and more. Its consistent hue reliably guarantees quality and esthetic functionality in the industries mentioned earlier. However, factors such as prevailing temperature, molar ratio, pH, stabilizer type, and solvent type should be checked to increase bioavailability efficiency. Enzymatic acylation and esterification are employed to increase the stability of the C3G molecule by altering its chemical structure to enhance non-therapeutic functions (68, 69). Improving bioaccessibility, bioavailability, and stability characteristics of the C3G molecule using green technologies will enhance its functions in therapeutic and non-therapeutic applications. Table 3 presents C3G food extracts, green technology adopted to improve their stability and bioavailability.

## 4 Therapeutic and non-therapeutic functions of the C3G molecule

### 4.1 Therapeutic mechanisms of the C3G molecule

The C3G molecule exerts its numerous multi-target organ biofunctions through the gut-microbiome-brain-liver-immune axis complex (see Figure 2). The gut–brain axis occurs directly or indirectly (via systemic circulation), with some gut microbes synthesizing neurotransmitters (i.e., *γ*-aminobutyric acid [GABA], noradrenaline, and dopamine) that target other organs. Essentially, the gut microbiota interacts with and affects organs involved with food metabolism (gut), transformation (liver), signaling (brain and nerve cells), and storage (adipose tissue). The gut communicates with the endocrine (cortisol), immune (cytokines), and neural (vagus, ENS, and spinal nerves) pathways (70). These interwoven multi-organ-system interactions have far-reaching health implications (14, 15, 70, 71). The C3G molecule’s interaction with the gut mucosal immune system is the core mechanism influencing host health status (31). The gut-microbiome environment consists of trillions of microorganisms, produces neurotransmitters, and communicates with the brain via the nervous and endocrine systems (72, 73). As a highly innervated organ, the gut microbiome forms a complex system known as the enteric nervous system. It contains immune cells known as gut-associated lymphoid tissue (GALT), which regulate immunity and inflammation.

In brief, gut microbes metabolize the C3G molecule, which is absorbed into the liver, influencing gene expression, enzyme activity, and lipid metabolism. Microbial populations in the gut secrete organic and short-chain fatty acids (SCFAs) that stimulate/communicate with the brain (14, 15). Postbiotics from gut microbiota activities influence the central nervous system (CNS) through the enteric and vagus nerves, the hypothalamic–pituitary–adrenal (HPA) axis, and microglia (14). The C3G molecule stimulates liver immune cells, such as Kupffer cells, to produce pro-inflammatory cytokines, including IL-6, TNF-*α*, and IL-1β; these cytokines alter the peripheral neural signaling and are released into the bloodstream, affecting CNS areas lacking blood–brain barriers (15). Additionally, the C3G molecule triggers the AMPK/Nrf-2 signal pathway, which increases the expression of detoxification and antioxidant enzymes, protecting the liver from harm and maintaining energy homeostasis in response to low energy levels by encouraging the breakdown of fatty acids and glucose to produce ATP (energy) (74). In addition, gut-metabolized C3G molecule acts directly on the brain by crossing the blood–brain barrier and influencing neurotransmitters and neural activity, or indirectly via the enteric nervous system and the vagus nerve. Table 1 shows examples of food-based C3G molecule sources, mechanisms, and a plethora of C3G health benefits, including anti-obesity (75, 76), anti-cancer (77), anti-microbial (78), antioxidative (79), cardio-protective (80), neuroprotective (81), anti-inflammatory (34), and improved reproductivity (82).

The ability of the C3G molecule to cross the blood–aqueous and blood–retinal barriers, accumulate in ocular tissues through glucose transporters, and absorb light in the UV (280–400 nm) and blue light (360–500 nm) regions is what makes it beneficial for eye health. The C3G molecule also mitigates photooxidation-induced apoptosis and angiogenesis in the retina by activating the Nrf2/HO-1 pathway and suppressing NF-*κ*B and its anti-apoptotic mechanisms (37). C3G-containing foods are linked to lower rates of cardiovascular disease due to their antioxidant properties, which counteract the effects of free radicals on cardiovascular health (37). The C3G molecule elicits synaptic plasticity and integrity, neurogenesis, blood–brain barrier crossing, modulation of cell signaling pathways, and gene expression in neuroinflammation (37). Although its neuroprotective processes are not fully understood, a study assessing the impact of C3G molecules on stroke patients stated that the molecule attaches to the TLR4, TLR7, and P2X4 receptors, reduces inflammation, and repairs damaged cells (83). It acts via the gut–brain axis, and while in the target tissue degrades in response to oxidative stress, producing metabolites that prevent ferroptosis and provide neuroprotection. Other works list the direct regulation of the microbial gut population, the production of metabolites (protocateic acid, vanillic acid, etc.), the induction of blood–brain barrier permeability by butyrate, activation of the PPARs—master regulator of energy metabolism, and the modulation of insulin-like growth factor-1 (a neuropeptide) (12, 35, 84, 85) as pathways through which the C3G molecule influences neurodegenerative diseases.

Oxidative damage and inflammation are two hallmarks that trigger cancer development and progression; the C3G molecule disrupts these harmful processes and curbs cancer via its anti-oxidative mechanism (37). By improving glucose metabolism through downregulating glucose transporters GLUT2 and increasing glucagon-like peptide-1 secretion, modulating hepatic lipid metabolism, downregulating the NF-*k*B pathway, and raising fecal butyric acid, the C3G molecule prevents diabetes and obesity by lowering inflammation and oxidative stress (37).

### 4.2 Non-therapeutic functions of the C3G molecule

The C3G molecule’s ability to promote health by preventing disease conditions additionally makes it a safe food additive of choice. Combining preservative (anti-microbial) and natural photochromic properties increases the desirability of the C3G molecule for specific industrial purposes (54, 86). Food colorants help reflect food quality by contributing to the desired food shade, which can suppress or stimulate the appetite (54). The search for natural dyes and colorants would make for the adoption of the C3G molecule, as legal restrictions, side effects, and health concerns de-market synthetic food dyes and colorants. Examples of currently used synthetic dyes and food colorants are indigo carmine, Allura red, and brilliant blue (5, 11, 28, 59, 63, 87). C3G is relatively stable with phytotherapeutic properties, making it a suitable colorant. With green stabilization improvement technologies, it will be increasingly utilized to enhance the appearance, appeal, and shelf life of food and drugs (pH < 2) (5, 57).

Foods packaged with biopolymers are a trendy solution adopted to curb the impact of using recalcitrant synthetic materials such as plastics (88). In addition, incorporating bioagents in “intelligent or smart packaging” helps detect changes in food storage conditions (89). Researchers used *Carrageenan* biopolymer and *Jaboticaba* peel extract (JPE) to create a smart food packaging film with biosensor features that could detect pH changes during storage (89). The anti-microbial activity of the C3G molecule can extend the shelf life and safety of packaged food items (when incorporated into packaging materials) (89–92). As a significant breakthrough in food technology, using the C3G molecules as an intelligent food packaging (IFP) component sets it apart, being non-toxic with antimicrobial properties. This curtails food toxicity concerns with other material types (93).

The C3G molecule is used as a bio-additive in cosmetic products such as hair dyes, make-up, sunscreens, nail colorants, skin and hair care products, and skin and hair cleansing products due to the high demand for organically derived extracts and products associated with a healthy aging outcome (37). Applying moisturizing gel incorporated with the C3G molecule protected mice’s skin against UVB-induced chronic photodamage (94).

Additionally, the high photochromic feature of the C3G molecule has been utilized in solar cell systems as an attachment to ions, which excites more electrons and injects them into the conduction band of the ions (37, 95). There are also dye-sensitized solar cells (DSSCs) where the C3G molecule is used to replace synthetic semiconductors (95, 96). As a pH-dependent molecule, the authors found that alkaline conditions produced higher efficiency, resulting in a power density of 0.0036 mW/cm^2^. Finally, a different study that used computational methods, the energy gap for C3G and C3R was 3.37919316 eV and 0.28792381 eV, respectively (97). Concluding that anthocyanin extracts from Gayo Arabica coffee husk components may be useful as possible photosensitizers in DSSC.

## 5 Future perspectives and conclusion

### 5.1 Future perspectives

Green technologies can contribute to the safe and quality applications of bioactive molecules. As a biomolecule with growing research and application prospects, the C3G molecule’s future in functional foods could evolve toward personalized nutrition, driven by advances in genomics and microbiomics (24, 25). With additional studies around gut modulation, innovative and tailored formulations could be geared toward curbing chronic conditions precisely via personalized treatments.

As a way forward, more studies on the C3G molecule will benefit from health system connections made possible by *in vitro*, organ-on-chip micro-physiological models, multi-omics, and *in silico* computational models. Organ-on-a-chip (Ooc) technology is fast replacing traditional preclinical drug testing models as 2D cell culture and animal models are becoming limited and less predictable (98). Trials, incorporating the C3G molecule in (Organ-on-a-Chip) technology, would require meticulous planning and fabrication moving forward. The end goal would be to use necessary resources to replicate key (especially stomach, gut, and brain) organ functions and support disease modeling, drug discovery, and personalized medicine. For example, a study described by Cassotta et al. (99) mimicked the intestinal epithelial layer by using immune responsive cells (human peripheral blood monocytic cell line and its induced macrophages). They used curcumin and docosahexaenoic acid, known dietary supplements that modulate inflammation, and observed their anti-inflammatory effects through the downregulation of TNF-*α*, IL-6, IL-1*β*, and IL-10 cytokines. The C3G molecule has multi-organ targets and can be utilized in many similar research endeavors.

Artificial intelligence (AI) solutions for predicting mechanisms and appropriate dosages would be beneficial for C3G functional studies. A recent work employing computational strategies (network pharmacology and molecular docking) easily identified the C3G molecule as the most suited to alleviate *Bungarus multicinctus* (a snake) envenomation symptoms (100). As the world is gradually accepting products from genetically engineered microorganisms (GEMs) (48), producing functional food components through sustainable, innovative, and cost-effective means will curb the use of plant parts (especially for non-health functions) and help with food safety.

Finally, green biotechnologies utilizing plant-based bioactives will require robust safety regulations, and the integration of gut microbiota data with artificial intelligence offers data-driven, precision-based solutions for functional food development (101). However, regulatory challenges with certain green extraction technologies abound as these are relatively new processes, lacking established frameworks and guidelines (102). An example is the novel deep-eutectic solvents (DES), which, being in contact with food materials, need to undergo rigorous premarket safety assessments.

### 5.2 Conclusion

The C3G molecule is primarily explored due to its significant positive impact on gastrointestinal health and the reduction of chronic illness development. Despite its cheap and abundant plant-based source advantage, extracting the C3G molecule using conventional methods can negatively impact the environment and increase cost, thereby affecting its use for therapeutic and non-therapeutic functions. In addition, their low bioavailability and molecular instability due to several factors also limit their applications.

Green extraction and delivery technologies enhance bioaccessibility, bioavailability, and stability of the C3G molecule. These approaches, with less environmental impact, increased yield and uptake in the body and optimized the therapeutic functions while minimizing the destabilizing ecological/carbon footprint impact of conventional extraction methods. Green stabilization and delivery technologies enhance the quality and safe use of the C3G molecule as an effective bio-monitoring component for smart packaging, components of solar cells in energy generation, and safe natural colorants in foods, medicines, and cosmetic products.

Although nutrients are not consumed in isolation, understanding the mechanisms of the C3G molecule remains a key aspect of nutrition research, significantly contributing to the formulation of functional foods. In addition, its non-therapeutic photochromatic properties in many studies, such as a component for solar cells for energy generation, safe food ingredients, and smart packaging, make it stand out as a research-worthy food-sourced molecule in many C3G studies.
